# Supplementary material for: Comparative Analysis of Stk11/Lkb1 versus Pten Deficiency in Lung Adenocarcinoma Induced by CRISPR/Cas9
Source: Cancers (Basel). 2021 Feb 26;13(5):974. doi: 10.3390/cancers13050974 (PMC7956254; doi:10.3390/cancers13050974)
Supplement: Supplementary file 1 [file cancers-13-00974-s001.zip › supplementary/cancers-1053958-supplementary.pdf]

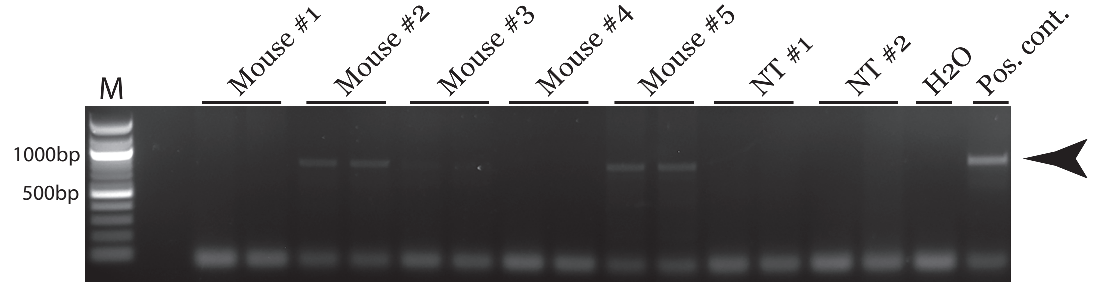

Figure S1: AAV DNA is present in the lung 3 weeks post viral delivery.

Presence of viral construct DNA in the lungs 21 days post-delivery. NT = non-treated negative control, Positive control = AAV_STK plasmid.

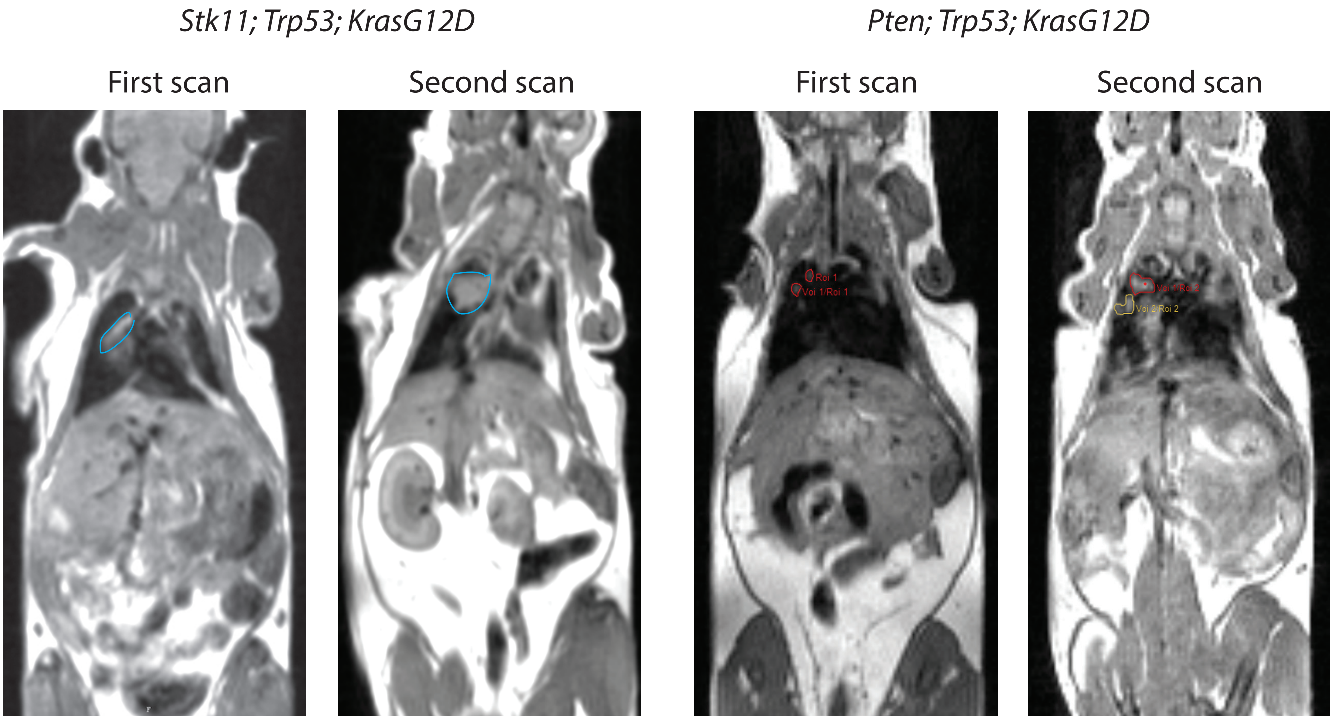

Figure S2: Sequentially scanning of lung tumors.

Mice were scanned multiple times during the cancer progression to monitor the tumor growth between the two experimental groups. Representative images are shown for two consecutively scans. The tumors are marked with a dotted line.

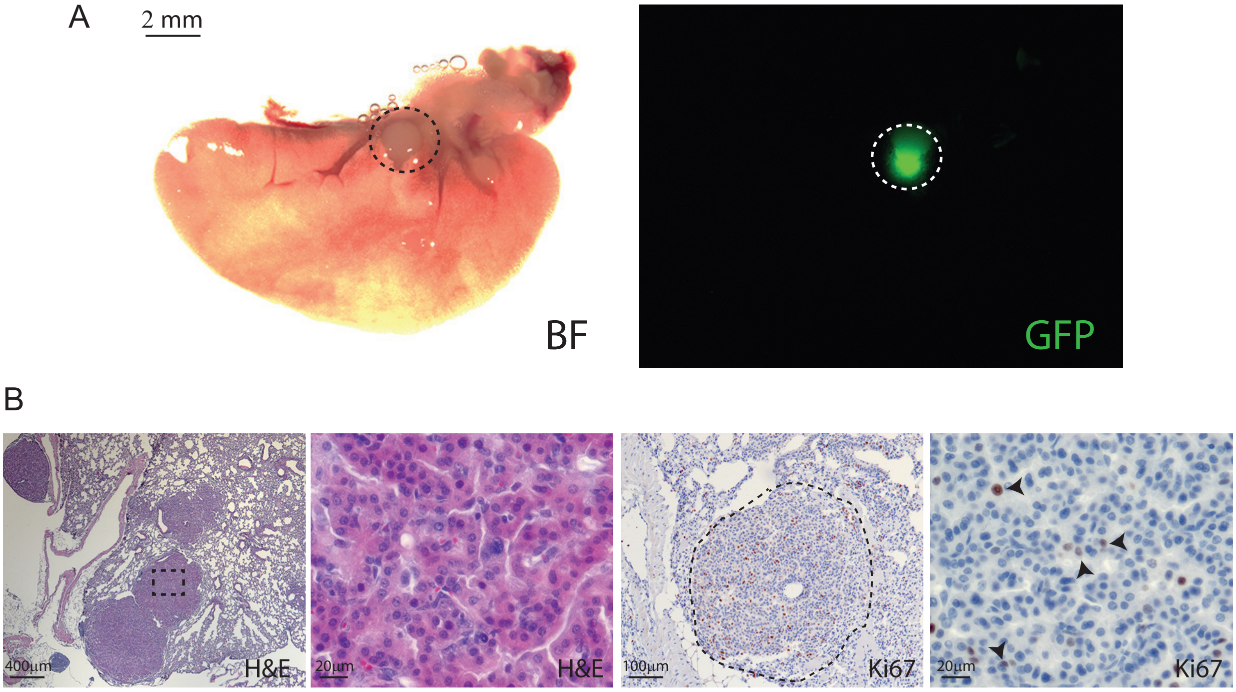

Figure S3: Development of lung tumor by loss of Trp53 and Kras^{G12D} mutation.

Mice inoculated with AAV particles containing sgRNAs for induction of *Trp53* and *Kras^{G12D}* mutations. A) Bright-field and GFP image of a lung lobe 6 months post-initiation. B) H&E and Ki67 stained paraffin section from lung samples 6 months after tumor initiation (n=3). Dotted box marks the area of magnification and dotted cycle marks the tumor. Representative pictures are shown.

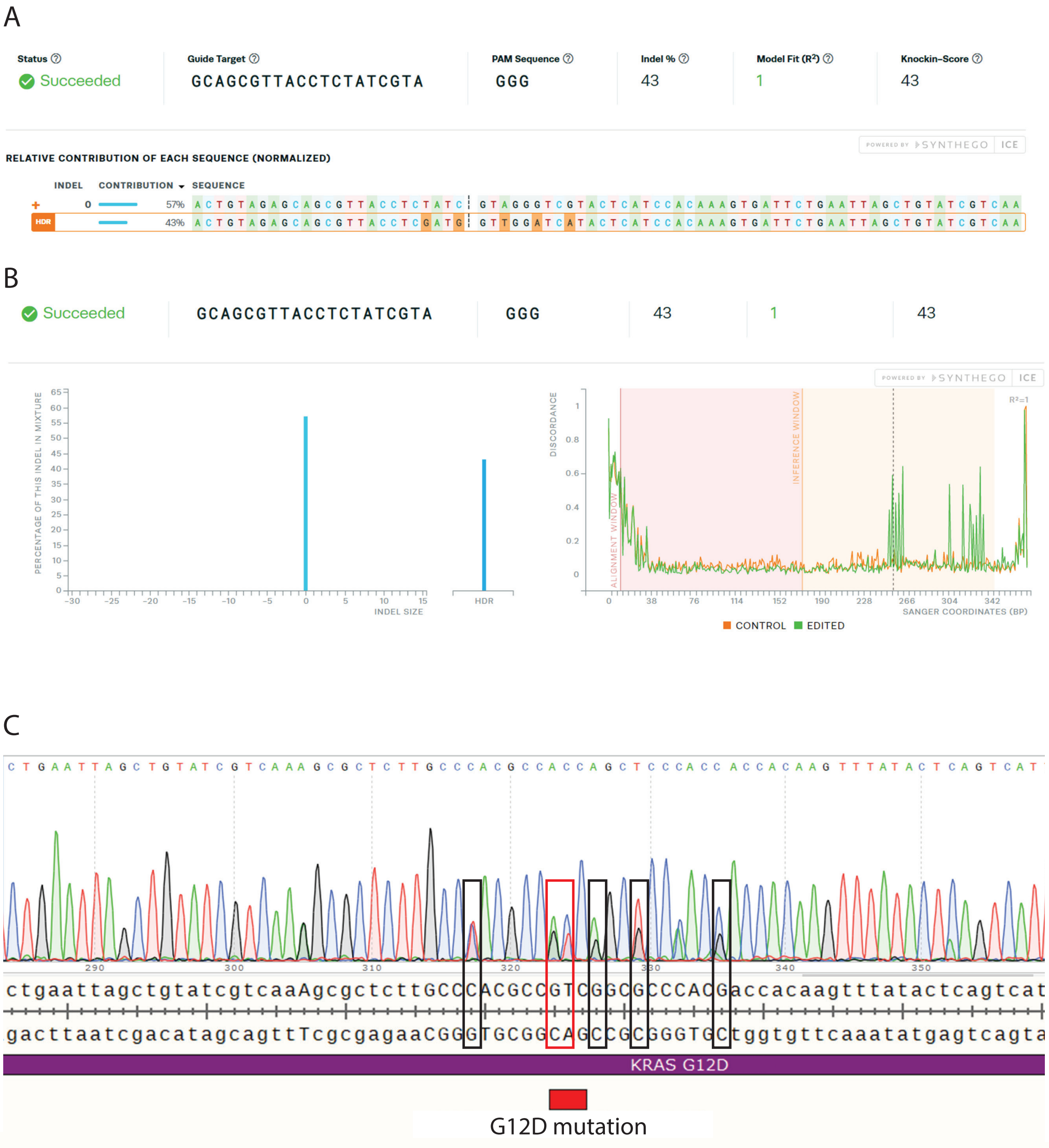

Figure S4: Detection of KrasG12D mutation in lung tumors induced by CRISPR and HDR.

Synthego ICE analysis was used to analyse KrasG12D mutations induced by CRISPR combined with a homology-directed repair template (HDR). **A)** Percentages of HDR mutated DNA in a lung tumor sample compared to wild type DNA. The HDR template introduces silent mutations in the binding site of the guide. In this sample, 43% of the DNA contain mutations induced by the HDR template. **B)** Insertion and deletion (Indel) distribution in the same sample showing peaks where mutations have been introduced by the HDR template. **C)** Alignment of base call from the analysis sample and the KrasG12D HDR template at the site of KrasG12D mutation. Black boxes mark silent mutations and the red box marks the KrasG12D mutation. A representative sample is shown.

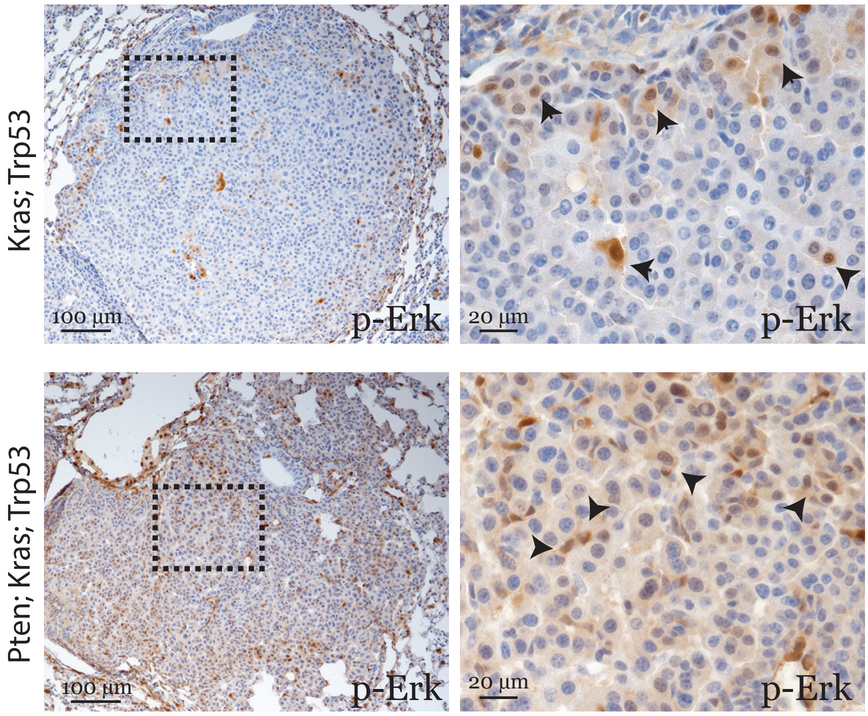

Figure S5: Increase levels of p-Erk in lung tumors.

Mice were inoculated with AAV particles containing sgRNAs for induction of *Trp53* and *Kras^{G12D}* mutations or *Pten, Trp53* and *Kras^{G12D}* mutations. Paraffin sections from lung samples 6 months after tumor initiation were stained for p-Erk (n=3). Dotted box marks the area of magnification and arrowheads mark positive cells. Representative pictures are shown.

Primer:	Sequence:
Pten_sgRNA_F	caccGCAGCAATTCACTGTAAAGC
Pten_sgRNA_R	aaacGCTTTACAGTGAATTGCTGC
Pten_seq_F	CTCCCTGGAGTGAAGAGCAC
Pten_seq_R	GTGTGCCTAGCACCTACTCC
Kras_seq_F	AGGCCTGCTGAAAATGACTGA
Kras_seq_R	CAAAGCACGGATGGCATCTT
Trp53_seq_F	ATAGAGACGCTGAGTCCGGT
Trp53_seq_R	CAAAGAGCGTTGGGCATGTG
Stk11_seq_F	CACCGAGGTAATCTACCAGCC
Stk11_seq_R	GCAGGCTGCCCTAACATACA
AAV_presence_F	GCAGCGTTACCTCTATCGTA
AAV_presence_R	GAGCCAATTCCCACTCCTTT
AAV_titration_F	GGGATCTTTGAGGCAACCCA
AAV_titration_R	TGACCTGACCAGGCGAGATA

Table S1: Primers and guides sequence.
