# Supplementary figures and images for: Comparative Analysis of Stk11/Lkb1 versus Pten Deficiency in Lung Adenocarcinoma Induced by CRISPR/Cas9
Source: Cancers (Basel). 2021 Feb 26;13(5):974. doi: 10.3390/cancers13050974 (PMC7956254; doi:10.3390/cancers13050974)

pAKT

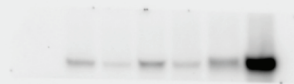

pERK

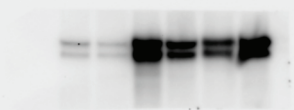

Actin

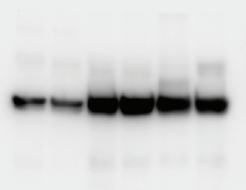

Supplement: Supplementary file 1 [file cancers-13-00974-s001.zip › supplementary/cancers-1053958-original-images.pdf]
